# Personal

**Published:** 1896-10

**Authors:** 


### Personal.

AT the recent meeting of the American Public Health Association, held at Buffalo, the United States was ably represented by Geo. M. Sternberg, M. D., Surgeon-General, U. S. Army ; Albert L. Gihon, M. D., Medical Director of the Navy (retired) ; Alfred A. Woodhull, M, D., Lieutenant-Colonel U. S. Army, who presided at the meetings and D. E. Salmon, D. V. M., Chief of the Bureau of Animal Industry, and others.

Among the prominent Canadian sanitarians there were present :

Dr. Fredrick Montizambert, of Quebec, General Superintendent of Quarantines of the Dominion of Canada; Dr. Peter H. Bryce, of Toronto, Secretary of the Provincial Board of Health of Ontario; and Dr. Wyatt Johnston, of Montreal, Bacteriologist of the Provincial Board of Health of Quebec.

---

While attending the annual meeting of the Association of Erie Railway Surgeons, held at the Kent House, Lakewood, September 21, 1896, Dr. John L. Eddy, of Olean, president of the association, met with quite a severe accident, falling into an open elevator shaft, down a distance of eight feet, sustaining a deep cut across the forehead, a badly sprained ankle and a severely bruised hip.

---

Drs. Harry A. Powers and Oscar H. Kraft, of Buffalo, who have been house surgeon and ambulance surgeon, respectively, at the Fitch hospital the past year, have opened an office at 47 West Swan street. Dr. Powers, hours: 9.30–11 a. m., 1.30–3 and 7 p. m. Dr. Kraft, hours: 8–9.30 a. m., 3–4.30 and 7 p. m. Telephone, Seneca 310.

---

At the one hundred and twenty-eighth annual commencement of Brown University the honorary degree of LL. D. was conferred on Dr. George M. Sternberg, of Washington, D. C., Surgeon-General of the United States Army.

---

Dr. William C. Fritz, of Buffalo, who has been on the invalid list, and under the care of Dr. Charles Cary for the last four months, has returned from the country and resumed his practice at 164 East North street.

---

Dr. Charles A. L. Reed has been elected gynecologist and abdominal surgeon on the staff of the Cincinnati Hospital, vice Dr. T. A. Reamy resigned.—*Columbus Medical Journal*, September.

---

Dr. William H. Humiston, of Cleveland, has been elected associate professor of gynecology in the medical department of Western Reserve University.

Dr. L. H. Staples, of Buffalo, has removed from 179 East Ferry street to 121 East Ferry street, corner Masten; office hours, 8 to 10 a. m., 1 to 3 and 7 to 8 p. m.

Dr. Ray H. Johnson and wife have returned from a three weeks' vacation to New York and the seashore.

Dr. George R. Stearns and daughter have returned from a two weeks' trip to the seashore and New England.

Dr. John Townsend Pitkins has returned from Georgian Bay.

